# The process of stopping recruitment and trial treatment in a trial of a psychological therapy for people with personality disorder following a safety alert

**DOI:** 10.1186/1745-6215-14-S1-P13

**Published:** 2013-11-29

**Authors:** Florence Day, Mary McMurran, Lelia Duley

**Affiliations:** 1 Nottingham Clinical Trials Unit, University of Nottingham, Nottingham, UK; 2 Institute of Mental Health, University of Nottingham, Nottingham, UK

## 

The study team were advised by the independent oversight committees to stop recruitment to the trial and the delivery of the trial therapy due to a difference in the number of reported hospitalisations between the treatment and control group.

There was a lack of guidance on how to proceed with implementation of the changes in a trial of this type and little precedent for cessation of trial treatment in a psychotherapy trial following a safety alert. Furthermore, there were concerns over the potential for distress and disruption to participants and clinical teams as a result of the trial changes and the potential risks of discontinuing treatment prematurely.

A collaborative approach to the development of the process and supporting documents was adopted, with attention given to the impact on participants and clinical teams. The changes were implemented as an urgent safety measure.

The following were developed:

• Written process for implementation.

• Written information for participants, potential participants and clinical teams.

• Guidance documents for sites.

• Arrangements for local and national telephone helplines.

• Media response.

The following key features were intended to reduce the impact on participants and clinical teams:

• Prompt, but careful action.

• Staged, allowing adequate time to inform and support participants and clinical teams, and ensuring feedback received could inform further development.

• Clinically appropriate, with reference to national guidelines and accepted good practice.

• Honest, concise and consistent information given to all concerned and follow-up support offered as appropriate.

• Regular review and reflection.

